# Novel specific activity-based probes validate KLK proteases as druggable targets

**DOI:** 10.1080/15384047.2022.2074775

**Published:** 2022-06-02

**Authors:** Georgia Sotiropoulou, Eleni Zingkou, Georgios Pampalakis

**Affiliations:** a Department of Pharmacy, School of Health Sciences, University of Patras, Rion-Patras, Greece; b Department of Pharmacology-Pharmacognosy, School of Pharmacy, Aristotle University of Thessaloniki, Thessaloniki, Greece

**Keywords:** activity-based probes, ABPs, kallikrein-related peptidases, KLKs, KLK-ABPs, KLK proteolytic cascades, activography, theranostic ABPs

Dear Editors

We read with great interest the recent publication by Lee et al.,^1^ in which the important paper by Lovell et al.^2^ is presented as the first report of activity-based protein profiling (ABPP) selective for individual KLKs in a complex biological context. Fairly, at the exact same time when the manuscript by Lovell et al.^2^ appeared, a novel KLK7-specific phosphonate activity-based probe (ABP) was reported, which could discriminate KLK7 from other active KLKs.^3^ That ABP was used to quantify the active KLK7 *in situ*, specifically, in the epidermis, in a pathological setting in which multiple KLK proteases are active and functionally implicated in disease.^3^ The KLK7-ABP was also modified to a quenched ABP (*q*ABP) to enable monitoring of KLK7 activity *in vivo*.^4^

Notably, the KLK7-ABP rescued major disease hallmarks, when applied onto the epidermis of *Spink5^−/−^Klk5^−/−^* mice.^3^ *Spink5^−/−^* mice represent an established preclinical model that recapitulates the overdesquamating and inflammatory skin disease named Netherton syndrome, a rare ichthyosis caused by inactivating mutations in *SPINK5* encoding the LEKTI inhibitor of serine proteases. It is well established that LEKTI deficiency causes unopposed (disease causative) proteolytic activities in the epidermis. In *Spink5^−/−^Klk5^−/−^, Klk5* was genetically ablated on the Netherton background to provide proof of concept for drugging the KLK5 activity for pharmacotherapy of Netherton syndrome and, potentially, other diseases like atopic dermatitis. The *Spink5^−/−^Klk5^−/−^* model proved that KLK5 represents a novel drug target.^5–7^ Nevertheless, inhibition of KLK5 is not sufficient for permanent rescue/therapy of this very severe (potentially lethal) disease, since delayed inflammation develops soon in these mice due to KLK7 activity.^5,7^ This was also revealed by the fact that inhibition with the KLK7-ABP attenuates inflammation.^3^ Thus, additional targeting of inflammation is required in *Spink5^−/−^Klk5^−/−^* with either KLK7 inhibitors^7^ or anti-TNFα biologics.^5^ Overall, these studies have provided a strong rational for drugging the KLK family and set the grounds for pharmaceutical development of the recently described boronate inhibitor for KLK5.^8^ For the first time, use of the KLK7-ABP (in combination with genetic knockout) allowed validation of the given KLK7 activity as a drug target in a well-established disease context *in vivo* and provided preclinical proof of concept for using ABPs targeting the KLKs as theranostics, namely dually exploitable for disease diagnosis and treatment. Previously, ABPs for cathepsins were used as theranostics and cancer imaging tools by the group of Galia Blum.^9,10^

Beyond these studies that established KLKs as druggable targets in skin pathologies, targeting KLKs for treatment of prostate cancer was the subject of studies performed more than a decade ago. Specifically, the prostate proteases KLK2 and KLK3 were exploited for activation of anticancer prodrugs developed for treatment of prostate cancer.^11^ Drugging KLKs for prostate cancer, for example, resulted in a boronic acid-based inhibitor of KLK3,^12^ while KLKs have been clinically tested by various approaches, and there have been success stories in this respect, PROSTVAC (an active immunotherapy vaccine that contains KLK3/PSA) being one such example.^13^ In contrast to the Lee et al.^1^ KLKs are druggable.^11–14^

Following mapping of the KLK gene cluster in 2000, extensive knowledge was accumulated on the abundance of immunoreactive KLKs in normal and cancer-related settings, revealing these enzymes as potential biomarkers and/or therapeutic targets for cancer but also other diseases,^14^ including viral infections of high current interest.^15^ This triggered attempts to quantify the active forms of KLK proteases in biological and clinical samples. Coordinated dysregulation of specific KLKs in disease states (including cancer, inflammation, and neurodegeneration) suggested that multiple KLK enzymes could interplay in the mode of “proteolytic cascades,” which could also crosstalk with proteases of differing specificities. It was proposed that specific regulatory KLK cascades could be functionally implicated in (patho)physiology in certain tissues like the skin and the prostate.^16^ Of note, a proteolytic cascade pathway involving KLK5 (and putatively other KLK proteases) was proposed to be implicated in prostate physiology (i.e., seminal clot liquefaction) and prostate cancer progression, originally by Michael et al.,^17^ more than 15 years ago.

In fact, the prime attempt to quantify the proportion of enzymatically active KLK6 relative to total immunoreactive KLK6 was in 2008, by Oikonomopoulou et al.,^18^ who developed an ELISA using an ABP that targets trypsin-like serine proteases, coupled to a capturing antibody for KLK6, to specifically monitor the active KLK6 in CSF, ascites fluid from ovarian cancer patients, and supernatants from cancer cell lines. It was suggested, then, that ABPs could increase the diagnostic potential of KLK6 over classical immunoassays that determine protein abundance. Following, a new phosphonofluoridate ABP for serine proteases was generated and used to develop “Activography,”^19^ a novel histology method for spatial localization and monitoring of enzyme activities in tissues. Activography is a sensitive, versatile, and easily adaptable method that could replace the technically demanding *in situ* zymography used to determine proteolytic activities in tissues.^19^ Indeed, activography proved valuable for spatial localization of the activity of KLK7 in skin biopsies obtained from patients with Netherton syndrome^3^ but also the overall epidermal proteolytic activities in various other skin diseases. Remarkably, de-regulated proteolysis was revealed to be a common hallmark in these pathologies of differing genetic causes.^20^ Progress in this direction will depend on the availability of specific ABPs to dissect individual protease activities in complex (patho)biological environments.

The first KLK-specific ABP (DKFZ-633) was reported in 2018 and was based on the addition of a 1-alkyne moiety on the KLK6-specific depsipeptide inhibitor DKFZ-251.^21^ DKFZ-633 enabled the detection of active KLK6 by click chemistry. Soon thereafter, the VRFR peptide substrate was found specific for KLK13 and was modified to an inhibitor by addition of a chloromethyl ketone warhead and to a KLK13-ABP by extra addition of a biotin detection tag at the *N-*terminus (biotin-PEG-VRFR-CMK).^22^ Later, a KLK14-specific phosphonate ABP was synthesized to monitor the activation of ectopically expressed KLK14 in LNCaP cells.^23^ Cumulatively, the KLK-ABPs reported by Lovell et al.^2^ have not marked the first application of ABPs capable to discriminate between individual KLK activities, as stated by Lee et al.^1^ Clearly, the Lovell et al. study represents a breakthrough forward as the first multiplex analysis of KLK activities, namely concurrent orthogonal determination of KLK2, KLK3, and KLK14 activities in prostate cancer cell lines, which revealed *in vitro* cross-activation events of KLK2, KLK3, and KLK14 zymogens.

Individual protease activities need to be interrogated in the frame of complex biological networks *in vivo* by use of protease-specific ABPs; however, multiplexed detection of enzyme activities is limited by the spectral overlap of commonly used fluorophores. In this direction, the study by Lovell et al.^2^ definitely constitutes a significant step of progress as it enabled parallel monitoring of multiple KLK activities. Multiplexing is indispensable for enzymes acting in the mode of cascade pathways like the KLK proteases; moreover, it allows monitoring of dynamic alterations of KLK activities in response to perturbations like, for example, drug treatments. Further, the KLK14-ABP revealed that this protease drives malignant prostate cell migration, a key step in progression of cancer metastasis. Previously, other KLKs, i.e., KLK5 and KLK7 had been validated as drug targets in skin inflammatory diseases in which the implication of KLK proteolytic cascades is well-defined *in vivo*.^24,25^

## References

[1] Lee KY, Chau CH, Price DK, Figg WD. Drugging the undruggable: activity-based protein profiling offers opportunities for targeting the KLK activome. Cancer Biol Ther. 2022;23(1):136–138. doi:10.1080/15384047.2022.2033059. 35129066 PMC8820805

[2] Lovell S, Zhang L, Kryza T, Neodo A, Bock N, De Vita E, Williams ED, Engelsberger E, Xu C, Bakker AT, et al. A suite of activity-based probes to dissect the KLK activome in drug-resistant prostate cancer. J Am Chem Soc. 2021;143(23):8911–8924. doi:10.1021/jacs.1c03950. 34085829 PMC9282638

[3] Bisyris E, Zingkou E, Kordopati GG, Matsoukas M, Magriotis PA, Pampalakis G, Sotiropoulou G. A novel theranostic activity-based probe targeting kallikrein 7 for the diagnosis and treatment of skin diseases. Chem Commun. 2021;57(53):6507–6510. doi:10.1039/d1cc01673c. 34105530

[4] Bisyris E, Zingkou E, Kordopati GG, Matsoukas M, Magriotis PA, Pampalakis G, Sotiropoulou G. Generation of a quenched phosphonate activity-based probe for labelling the active KLK7 protease. Org Biomol Chem. 2021;19(31):6834–6841. doi:10.1039/d1ob01273h. 34308939

[5] Zingkou E, Pampalakis G, Sotiropoulou G. Cocktails of KLK5 protease inhibitors and anti-TNFα therapeutics: an effective treatment for Netherton syndrome. J Clin Immunol. 2022;42(3):597–605. doi:10.1007/s10875-021-01195-0. 35040012

[6] Furio L, Pampalakis G, Michael IP, Nagy A, Sotiropoulou G, Hovnanian A. KLK5 inactivation reverses cutaneous hallmarks of Netherton syndrome. PLoS Genet. 2015;11(9):e1005389. doi:10.1371/journal.pgen.1005389. 26390218 PMC4577096

[7] Kasparek P, Ileninova Z, Zbodakova O, Kanchev I, Benada O, Chalupsky K, Brattsand M, Beck IM, Sedlacek R. KLK5 and KLK7 ablation fully rescues lethality of Netherton syndrome-like phenotype. PLoS Genet. 2017; 13(1):e1006566. doi:10.1371/journal.pgen.1006566. 28095415 PMC5283769

[8] Liddle J, Beneton V, Benson M, Bingham R, Bouillot A, Boullay AB, Brook E, Cryan J, Denis A, Edgar E, et al. A potent and selective kallikrein-5 inhibitor delivers high pharmacological activity in skin from patients with Netherton syndrome. J Invest Dermatol. 2021;141(9):2272–2279. doi:10.1016/j.jid.2021.01.029. 33744298

[9] Ben-Nun Y, Merquiol E, Brandis A, Turk B, Scherz A, Blum G. Photodynamic quenched cathepsin activity-based probes for cancer detection and macrophage targeted therapy. Theranostics. 2015;5(8):847–862. doi:10.7150/thno.10854. 26000057 PMC4440442

[10] Weiss-Sadan T, Ben-Nun Y, Maimoun D, Merquiol E, Abd-Elrahman I, Gotsman I, Blum G. A theranostic activity-based probe for noninvasive intervention in cardiovascular diseases. Theranostics. 2019;9(20):5731–5738. doi:10.7150/thno.34402. 31534515 PMC6735363

[11] Hannu K, Johanna M, Ulf-Håkan S. KLK-targeted therapies for prostate cancer. Ejiff. 2014;25(2):207–218. PMC4975297 27683469

[12] LeBeau AM, Singh P, Isaacs JT, Denmeade SR. Potent and selective peptidyl boronic acid inhibitors of the serine protease prostate-specific antigen. Chem Biol. 2008;15(7):665–674. doi:10.1016/j.chembiol.2008.05.020. 18635003 PMC3360951

[13] Kantoff PW, Schuetz TJ, Blumenstein BA, Glode LM, Bilhartz DL, Wyand M, Manson K, Panicali DL, Laus R, Schlom J, et al. . Overall survival analysis of a phase II randomized controlled trial of a Poxviral-based PSA-targeted immunotherapy in metastatic castration-resistant prostate cancer. J Clin Oncol. 2010;28(7):1099–1105. doi:10.1200/JCO.2009.25.0597. 20100959 PMC2834462

[14] Sotiropoulou G, Pampalakis G. Targeting the kallikrein-related peptidases for drug development. Trends Pharmacol Sci. 2012;33(12):623–634. doi:10.1016/j.tips.2012.09.005. 23089221

[15] Pampalakis G, Zingkou E, Panagiotis C, Sotiropoulou G. Kallikreins emerge as new regulators of viral infections. Cell Mol Life Sci. 2021;78(21–22):6735–6744. doi:10.1007/s00018-021-03922-7. 34459952 PMC8404027

[16] Pampalakis G, Sotiropoulou G. . Tissue kallikrein proteolytic cascade pathways in normal physiology and cancer. Biochim Biophys Acta-Rev Cancer. 2007;1776(1):22–31. doi:10.1016/j.bbcan.2007.06.001. 17629406

[17] Michael IP, Pampalakis G, Mikolajczyk SD, Malm J, Sotiropoulou G, Diamandis EP. Human tissue kallikrein 5 is a member of a proteolytic cascade pathway involved in seminal clot liquefaction and potentially in prostate cancer progression. J Biol Chem. 2006;281(18):12743–12750. doi:10.1074/jbc.M600326200. 16517595

[18] Oikonomopoulou K, Hansen KK, Baruch A, Hollenberg MD, Diamandis EP. Immunofluorometric activity-based probe analysis of active KLK6 in biological fluids. Biol Chem. 2008;389(6):747–756. doi:10.1515/BC.2008.086. 18627291

[19] Pampalakis G, Zingkou E, Vekrellis K, Sotiropoulou G. “Activography”: a novel, versatile and easily adaptable method for monitoring enzymatic activities *in situ*. Chem Commun. 2017;53(22):3246–3248. doi:10.1039/c7cc01081h. 28256664

[20] Zingkou E, Pampalakis G, Kiritsi D, Valari M, Jonca N, Sotiropoulou G. Activography reveals aberrant proteolysis in desquamation diseases of differing genetic backgrounds. Exp Dermatol. 2019;28(1):86–89. doi:10.1111/exd.13832. 30390391

[21] De Vita E, Schüler P, Lovell S, Lohbeck J, Kullmann S, Rabinovich E, Sananes A, Heßling B, Hamon V, Papo N, et al. . Depsipeptides featuring a neutral P1 are potent inhibitors of kallikrein-related peptidase 6 with on-target cellular activity. J Med Chem. 2018;61(19):8859–8874. doi:10.1021/acs.jmedchem.8b01106. 30212625

[22] Gruba N, Bielecka W, Wysocka M, Wojtysiak A, Brzezińska-Bodal M, Sychowska K, Kalińska M, Magoch M, Pęcak A, Falkowski K, et al. Development of chemical tools to monitor human kallikrein 13 (KLK13) activity. Int J Mol Sci. 2019;20(7):1557. doi:10.3390/ijms20071557. 30925705 PMC6479877

[23] Kryza T, Bock N, Lovell S, Rockstroh A, Lehman ML, Lesner A, Panchadsaram J, Silva LM, Srinivasan S, Snell CE, et al. The molecular function of kallikrein-related peptidase 14 demonstrates a key modulatory role in advanced prostate cancer. Mol Oncol. 2020;14(1):105–128. doi:10.1002/1878-0261.12587. 31630475 PMC6944120

[24] Sotiropoulou G, Zingkou E, Pampalakis G. . Reconstructing the epidermal proteolytic cascades in health and disease. J Pathol. 2022;in press. doi:10.1002/path.5888. 35218558

[25] Sotiropoulou G, Zingkou E, Bisyris E, Pampalakis G. . Activity-based probes for proteases pave the way to theranostic applications. Pharmaceutics. 2022;14(5):977. doi:10.3390/pharmaceutics14050977. 35631563 PMC9145445

